# Simulating Dynamics of Circulation in the Awake State and Different Stages of Sleep Using Non-autonomous Mathematical Model With Time Delay

**DOI:** 10.3389/fphys.2020.612787

**Published:** 2021-01-13

**Authors:** Anatoly S. Karavaev, Yurii M. Ishbulatov, Mikhail D. Prokhorov, Vladimir I. Ponomarenko, Anton R. Kiselev, Anastasiia E. Runnova, Alexey N. Hramkov, Oxana V. Semyachkina-Glushkovskaya, Jürgen Kurths, Thomas Penzel

**Affiliations:** ^1^Saratov Branch of the Institute of Radio Engineering and Electronics of Russian Academy of Sciences, Saratov, Russia; ^2^Smart Sleep Laboratory, Saratov State University, Saratov, Russia; ^3^Department of Innovative Cardiological Information Technology, Saratov State Medical University, Saratov, Russia; ^4^Physics Department, Humboldt University of Berlin, Berlin, Germany; ^5^Research Department Complexity Science, Potsdam Institute for Climate Impact Research (PIK), Potsdam, Germany; ^6^Interdisciplinary Sleep Medicine Center, Charité – Universitätsmedizin Berlin, Berlin, Germany

**Keywords:** mathematical modeling, cardio-vascular system, sleep studies, autonomic control, non-linear dynamics

## Abstract

We propose a mathematical model of the human cardiovascular system. The model allows one to simulate the main heart rate, its variability under the influence of the autonomic nervous system, breathing process, and oscillations of blood pressure. For the first time, the model takes into account the activity of the cerebral cortex structures that modulate the autonomic control loops of blood circulation in the awake state and in various stages of sleep. The adequacy of the model is demonstrated by comparing its time series with experimental records of healthy subjects in the SIESTA database. The proposed model can become a useful tool for studying the characteristics of the cardiovascular system dynamics during sleep.

## Introduction

The study of the dynamics of the circulatory system during sleep attracts a lot of attention (de Zambotti et al., 2018; Tall and Jelic, 2019; Kontos et al., 2020; Nano et al., 2020). It was shown that scaling properties of the cardiac dynamics are different during sleep and wake periods (Ivanov et al., 1999). Characteristics of rapid eye movement (REM) sleep are correlated with exacerbation of ischemic heart disease, sometimes even leading to myocardial infarction (Schumann et al., 2010). These events are most common in patients with coronary artery disease (Nowlin et al., 1965). Of particular importance is the change in the dynamics of the loops of the autonomous control of blood circulation, due to the influence of the higher nervous centers on them (van Roon et al., 2004; Ivanov, 2006). In experimental studies (Nowlin et al., 1965; King et al., 1973), it was shown that in animals with aortic stenosis, sympathetic activation can lead to decreased myocardial perfusion. The development of pathologies such as apnea is associated with dysfunction of the autonomic control of the cardiovascular system (Aydin et al., 2004; Khoo and Blasi, 2013; Roder et al., 2018).

Despite numerous studies, the functioning of the cardiovascular system (CVS) during sleep is essentially *terra incognito*. This is due to the complexity of CVS, which includes a large number of interacting non-linear elements, as well as technical difficulties and ethical limitations of experimental studies. Therefore, the development of mathematical models based on “first principles,” i.e., using physical and physiological laws, is of great importance (Wolkenhauer, 2014). Such models allow one to simulate complex CVS dynamics and generate stationary time series of any length (Karavaev et al., 2019). Using models, it is possible to simulate various pathologies (Karavaev et al., 2016), drug effects (Karavaev et al., 2016), physiological tests (Ishbulatov et al., 2020), changes in physiological conditions (van Roon et al., 2004), and the influences of brain activity (van Roon et al., 2004) and respiration (Wessel et al., 2009; Cheng et al., 2010). The development of such models is promising for solving the problems of personalized medicine, when some parameters of the model can be estimated directly from the experimental data of a particular patient. The use of personalized mathematical models expands the possibilities of medical diagnostics and therapy, making it possible to predict the course of diseases and simulate the patient’s response to medications.

A number of mathematical models of CVS are known, which take into account the dynamics of the loops of autonomic control of blood circulation (Guyton et al., 1972; Ivanov et al., 1998; Seidel and Herzel, 1998; Ursino, 1998; Ottesen, 2000; Kotani et al., 2005). However, only a few studies are aimed at modeling the dynamics of autonomic control loops and CVS during sleep (van Roon et al., 2004). The most well-known model of CVS during sleep is PNEUMA (Cheng et al., 2010). It is the eleventh-order system, which contains more than 80 algebraic terms and more than 200 parameters, most of which are estimated empirically and have no physical or physiological meaning. The PNEUMA model has shown its adequacy and importance (Khoo et al., 2013). However, its complexity complicates the interpretation of the results. Moreover, the model is too cumbersome to be personalized and fitted to a particular patient.

A number of studies suggest that adequate simulation of CVS dynamics in different stages of sleep requires taking into account the effects associated with autonomic control. In experimental studies, it was shown that non-REM sleep (especially stage 4) is characterized by a decreased tone of sympathetic activity, a decrease in heart rate and average level of blood pressure, and a decrease of blood pressure variability (Sommers et al., 1993). Other studies reported pronounced respiratory sinus arrhythmia caused by parasympathetic activity (Zemaityte et al., 1984).

Rapid eye movement sleep is characterized by more complex dynamics, for which epochs of sharp increase in sympathetic activity, alternating with intervals of decreased sympathetic activity, are typical (Sommers et al., 1993). As shown by direct measurements from sympathetic nerves, on average, there is an increase in sympathetic activity, leading to an increase in blood pressure variability, but the heart rate corresponds to a waking person at rest. These results were confirmed in active experiments with blocking of sympathetic or parasympathetic autonomic control, which compensated for the corresponding changes in the dynamics of CVS (Zemaityte et al., 1984).

It is known that the transition to sleep is associated with the activity of the higher nervous system. In model studies (van Roon et al., 2004) and (Cheng et al., 2010), potential ways for the influence of higher nerve centers on autonomic control during sleep are presented.

In the present paper, we propose a more compact model describing the dynamics of CVS in different stages of sleep and during wakefulness. In contrast to our earlier models, the new model for the first time takes into account the effect of the cerebral cortex on blood circulation in the awake state and in sleep. The proposed model consists of four differential equations with time delay. The model has 55 parameters, 39 of which have physiological meaning and can be estimated experimentally. Despite the relatively simple structure, the model takes into account the non-linear properties of the autonomic control and simulates with good accuracy the time series of real arterial blood pressure and interbeat intervals of healthy resting subjects. As well as our earlier models, it reproduces the pathological changes that lead to arterial hypertension (Karavaev et al., 2016), reaction to the passive orthostatic test (Ishbulatov et al., 2018), and reaction to the autonomic blockade due to administration of Arfonad (Karavaev et al., 2016). Moreover, the model simulates the chaotic dynamics of heart rate (Karavaev et al., 2019) and phase synchronization between the autonomic control loops (Ishbulatov et al., 2017), which is observed in humans (Bartsch et al., 2012) and is important for diagnostics and understanding of some circulatory diseases (Prokhorov et al., 2003; Kiselev et al., 2016a,b, 2020).

The paper is devoted to the description of the proposed model and the study of its dynamics in the awaking state, during REM sleep, and during non-REM sleep. We also compared the simulated data with the experimental data from the known SIESTA database (Klösch et al., 2001).

## Materials and Methods

### Study Participants

Our study included recording of 20 healthy subjects that fulfilled the following criteria: no regular shift work, usual bedtime before midnight, and no acute depressive or anxious symptoms. Each subject was monitored by wrist-worn actigraphs (Actiwatch, Cambridge Neurotechnology, England) one-week prior and one-week after the recording session. Quality control ensured that there are no outliers in the final data set, and all signals were recorded with no protocol violations.

For each subject, we analyzed a set of three 20-minute ECG signals, one recorded in the waking state, one during REM sleep, and one during stage 4 of non-REM sleep. Each signal was recorded at the sampling rate of 200 Hz. The high-pass cutoff frequency was set between 1.6 Hz and 16 Hz.

### Mathematical Model

**FIGURE 1:**
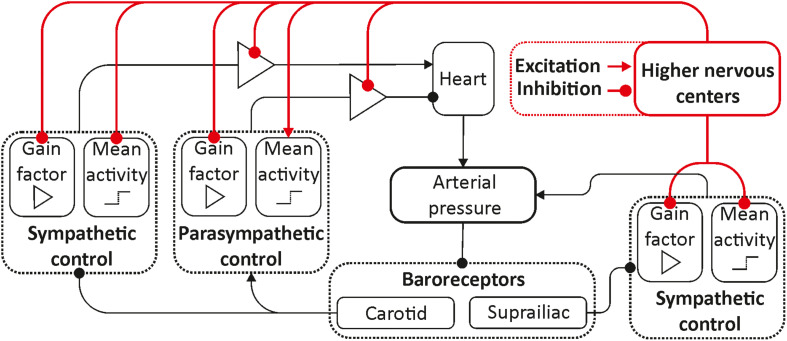
Structure of the model. Elements of the autonomic control are shown in black. Red color shows the inputs from the higher nervous centers.

To modify the previously proposed model (Karavaev et al., 2019), we used the ideas proposed in Cheng et al. (2010) and van Roon et al. (2004). In the equations of autonomic control of blood circulation, we added eight parameters characterizing the influence of the higher nervous centers. These parameters were set equal to zero for the awake state, but they took non-zero values during REM and non-REM sleep in order to simulate the increase and decrease in autonomic control activity. Figure 1 depicts the scheme of the model, where the places where the parameters are added are shown in red.

We used integrate-and-fire model to simulate the heart rate:

(1) $$\frac{d\,\varphi \,\left(t\right)}{d\,t}=\frac{1}{\left(T_{0}+\xi \right)}\,f_{s}\,\left(t\right)\,f_{p}\,\left(t\right),$$

where φ(*t*) is the phase of the sinoatrial node, *T*_0_ = 1.5 s is the heart rate, *f*_*s*_(*t*) and *f*_*p*_(*t*) are the sympathetic and parasympathetic factors, respectively, which modulate *T*_*0*_, and ξ is the 1/*f* noise (Ivanov et al., 1998), which is added to simulate myocardial and humoral control. Spectral properties of noise were chosen to match experimental signals (Bezerianos et al., 1995; Goldberger, 1996). In the absence of noise or autonomic control, the sinoatrial node generates periodic saw-like impulses with the period *T*_*0*_.

Within the first *T*_*sys*_ seconds (0.125 s) after the initiation of heart cycle, the arterial pressure rapidly grows and is described by the following equation:

(2) $$p_{s\,y\,s}\,\left(t\right)=D_{i-1}+S\,\left(t\right)\,\frac{\left(t_{i}-T_{i-1}\right)}{T_{s\,y\,s}}\,\exp\left(\frac{\left(t_{i}-T_{i-1}\right)}{T_{s\,y\,s}}\right)+k_{p}^{B}\,B\,\left(t\right),$$

where *D*_*i–1*_ is the systolic pressure at the end of the previous cardiac cycle, *T*_*i–1*_ is the moment of time when the previous cardiac cycle ended, *T*_*sys*_ is the duration of the current systolic phase, *t*_*i*_ is the time since the beginning of the current cardiac cycle, *B*(*t*) is the respiratory signal, $k_{p}^{B}$ is a non-dimensional amplitude of respiration, and *S*(*t*) is the cardiac contractility (Wessel et al., 2009) defined as follows:

(3) $$S\,\left(t\right)=S^{\prime }\,\left(t\right)+\left(\hat{S}-S^{\prime }\,\left(t\right)\right)\,\frac{S^{\prime ,n_{c}}\,\left(t\right)}{{\hat{S}}^{n_{c}}+S^{\prime ,n_{c}}\,\left(t\right)},$$

where $\hat{S}$ is the resting heart contractility, *n*_*c*_ is a fitting parameter, and $S^{\prime }\,\left(t\right)=S_{0}+k_{S}^{c}\,c_{c}\,\left(t\right)+k_{S}^{v}\,c_{v}\,\left(t\right)+k_{S}^{t}\,L_{i-1}$, where *S*_*0*_ is the contractility of the denervated heart, *c*_*c*_(*t*) is the concentration of noradrenaline in blood that circulates in the heart, *c*_*v*_(*t*) is the concentration of noradrenaline in vessels, $k_{S}^{c}$ and $k_{S}^{v}$ are the parameters that characterize sensitivity of contractility to changes in the noradrenaline concentration, $k_{S}^{t}$ is the parameter that characterizes sensitivity of contractility to changes of the heart rate, and *L*_*i–1*_ is the duration of the previous cardiac cycle. The values of all model parameters used for simulation are presented in Table 1.

**TABLE 1:** Parameters of the model and their description.

**Parameter**		**Value**		**Interpretation**				
**Physiologically interpretable parameters**								
*T*_*0*_		1.1 s		Heart period of the denervated heart				
σ^2^(ξ)		0.02 s^2^		Variance of unaccounted factors (humoral regulation, etc.) affecting heart rate variability				
*T*_*sys*_		0.02 s^2^		Duration of the systolic phase of the cardiac cycle				
$\hat{S}$		40 mm Hg		Resting heart contractility				
*T*_*br*_		3.57 s		Mean respiratory period				
*S*_*0*_		−13.8 mm Hg		Contractility of the denervated heart				
$k_{S}^{c}$		10 mm Hg		Sensitivity of contractility to changes of the noradrenaline concentration in the cardiac muscle				
$k_{S}^{v}$		20 mm Hg		Sensitivity of contractility to changes of the noradrenaline concentration in the vessel wall				
$k_{S}^{t}$		45 mm Hg × s^–1^		Sensitivity of contractility to changes in the heart rate				
*R_0_C*		2.0 s		Time constant for the peripheral vessels				
*a*_*s*_		−1		Parameters of the non-linear transfer function in the feedback loops of the baroreflex control				
*b*_*s*_		0.44						
*y*_*s*_		−0.25						
$a_{s}^{l}$		−1						
$b_{s}^{l}$		0.44						
$y_{s}^{l}$		−0.25						
τ_*c*_		2 s		Time constants for adrenaline concentration				
τ_*v*_		2 s						
σ^2^(*ζ*)		0.3 s^2^		Variance of the instantaneous respiration period				
*p*_*0*_		50 mm Hg		Thresholds of minimal blood pressure for excitation baroreflex response				
$p_{0}^{l}$		50 mm Hg						
*k*_*1*_		0.05 mm Hg^–1^		Arterial baroreceptor sensitivity				
*k*_*2*_		0.001 s × mm Hg^–1^						
$k_{1}^{l}$		0.05 mm Hg^–1^						
$k_{2}^{l}$		0.001 s × mm Hg^–1^						
θ_*v*_		2.5 s		Time delays associated with noradrenalin production				
θ_*c*_		1.5 s						
θ_*p*_		0.25 s		Time delays associated with acetylcholine production				
$v_{s}^{l\,0}$		4		Resting afferent tone of vascular tone sympathetic control				
$v_{s}^{0}$		4		Resting afferent tone of heart rate sympathetic control				
$v_{p}^{0}$		−0.5		Resting afferent tone of heart rate parasympathetic control				

	**Awake**		**REM sleep**	**Non-REM sleep**		**Awake**	**REM sleep**	**Non-REM sleep**

**Parameters that reflect the inputs of the higher nervous centers in awake state and sleep**								
$C_{s}^{b}$	0		−0.015	−0.01	$C_{p}^{v}$	0	0.2	0.6
$C_{s}^{l\,b}$	0		−0.015	−0.01	$C_{p}^{k}$	0	−0.09	−0.19
$C_{s}^{v}$	0		−0.1	−0.5	$C_{\varphi }^{s}$	0	−0.2	−0.2
$C_{s}^{l\,v}$	0		−0.1	−0.5	$C_{\varphi }^{p}$	0	−0.15	0
**Physiologically non-interpretable parameters for model fitting**								
$k_{p}^{B}$	4		$k_{s}^{l\,r}$	0.1	$k_{v}^{S}$	0.52	*n*_*s*_	2
*n*_*c*_	2.5		$k_{p}^{b}$	0.44	*k*_*v*_	0.2	$k_{\varphi }^{p}$	3.75
$k_{R}^{v}$	1.2		$k_{p}^{r}$	0.1	$k_{\varphi }^{c}$	3.7	${\hat{v}}_{p}$	2.5
$k_{s}^{r}$	0.1		$k_{c}^{S}$	0.04	${\hat{c}}_{c}$	2	*n*_*p*_	2

Respiratory signal *B*(*t*) is defined as follows:

(4) $$B\,\left(t\right)=\sin\left(\frac{2\,\pi \,t}{T_{b\,r}+\zeta }\right)$$

where *T*_*br*_ is the mean duration of respiratory cycle and ζ is a non-correlated Gaussian zero-mean noise that is used to modulate the rate of respiration after each cycle.

After *T*_*sys*_ seconds, the systolic phase of cardiac cycle ends and arterial pressure begins to slowly decrease in accordance with the Windkessel model (Frank, 1899):

(5) $$\frac{d\,p_{d\,i\,a}\,\left(t\right)}{d\,t}=-\frac{p_{d\,i\,a}\,\left(t\right)}{R\,\left(t\right)\,C},$$

where *C* is the parameter that reflects elastic properties of aorta and *R*(*t*) is the peripheral resistance, which is modulated by the sympathetic activity:

(6) $$R\,\left(t\right)=R_{0}\,\left(1+k_{R}^{v}\,c_{v}\,\left(t\right)\right),$$

where *R*_*0*_ is the resistance of unstressed vessels and $k_{R}^{v}$ is the parameter that describes sensitivity of peripheral resistance to changes of noradrenaline concentration in vessels walls *c*_*v*_(*t*).

The absolute value and rate of change of arterial pressure (Warner, 1958) are sensed by two baroreceptor nodes located in carotid sinus and in the major vessels of the lower body. Activities of these baroreceptor nodes [*v*_*b*_(*t*) and $v_{b}^{l}\,\left(t\right)$, respectively] are defined as follows:

(7) $$v_{b}\,\left(t\right)=k_{1}\,\left(p\,\left(t\right)-p_{0}\right)+k_{2}\,\frac{d\,p\,\left(t\right)}{d\,t},$$

(8) $$v_{b}^{l}\,\left(t\right)=k_{1}^{l}\,\left(p\,\left(t\right)-p_{0}^{l}\right)+k_{2}^{l}\,\frac{d\,p\,\left(t\right)}{d\,t},$$

where *p*_*0*_ and $p_{0}^{l}$ are the lowest pressure the baroreceptors react to and *k*_*1*_, *k*_*2*_, $k_{1}^{l}$, and $k_{2}^{l}$ are the sensitivity of arterial baroreceptors to arterial blood pressure and the rate of its change.

The higher nervous centers process the inputs of baroreceptors and adjust the activity of heart rate autonomic control *v*_*s*_(*t*) and vessel tone autonomic control $v_{s}^{l}\,\left(t\right)$. To describe their activity, we used equations with time delay and sigmoidal non-linearities as in Ringwood and Malpas (2000):

(9) $$v_{s}\,\left(t\right)=a_{s}\,\text{th}\,\left(\left(b_{s}+C_{s}^{b}\right)\,\left(v_{b}\,\left(t\right)\right)-C_{s}^{v}-v_{s}^{0}\right)+y_{s}+k_{s}^{r}\,B\,\left(t\right),$$

(10) $$v_{s}^{l}\,\left(t\right)=a_{s}^{l}\,\text{th}\,\left(\left(b_{s}^{l}+C_{s}^{l\,b}\right)\,\left(v_{b}^{l}\,\left(t\right)\right)-C_{s}^{l\,v}-v_{s}^{l\,0}\right)+y_{s}^{l}+k_{s}^{l\,r}\,B\,\left(t\right),$$

where $C_{s}^{b}$, $C_{s}^{l\,b}$, $C_{s}^{v}$, and $C_{s}^{l\,v}$ are the parameters, which characterize the influence of higher nervous centers, *a*_*s*_, *b*_*s*_, *y*_*s*_, $a_{s}^{l}$, $b_{s}^{l}$, and $y_{s}^{l}$ are the parameters of the non-linear transfer function in the feedback loops of the baroreflex control, $v_{s}^{0}$ is the resting afferent tone of the heart rate sympathetic control, $v_{s}^{l\,0}$ is the resting afferent tone of the vascular tone sympathetic control, and $k_{s}^{r}$ and $k_{s}^{l\,r}$ are the parameters characterizing the influence of respiration. Activity of parasympathetic autonomic control *v*_*p*_(*t*) is described as follows:

(11) $$v_{p}\,\left(t\right)=\max\left(0,C_{p}^{v}+v_{p}^{0}+\left(C_{p}^{k}+k_{p}^{b}\right)\,v_{b}\,\left(t\right)+k_{p}^{r}\,\left|B\,\left(t\right)\right|\right),$$

where $v_{s}^{0}$, $v_{s}^{l\,0}$, and $v_{p}^{0}$ are the sympathetic and parasympathetic activity under resting conditions, $C_{p}^{v}$ and $C_{p}^{k}$ are the parameters that reflect the influence of higher nervous centers on the parasympathetic control of heart rate, $k_{p}^{b}$ characterizes the influence of baroreflex activity, and $k_{p}^{r}$ characterizes the influence of respiration. Changes in the levels of sympathetic activation affect cardiac concentration of noradrenaline *c*_*c*_(*t*) and vascular concentration of noradrenaline *c*_*v*_(*t*):

(12) $$\frac{d\,c_{c}\,\left(t\right)}{d\,t}=-\frac{c_{c}\,\left(t\right)}{\tau_{c}}+k_{c}^{S}\,v_{s}\,\left(t-\theta_{c}\right),$$

(13) $$\frac{d\,c_{v}\,\left(t\right)}{d\,t}=-\frac{c_{v}\,\left(t\right)}{\tau_{v}}+k_{v}^{S}\,\left(v_{s}^{l}\,\left(t-\theta_{v}\right)+k_{v}\right),$$

where τ_*c*_ and τ_*v*_ are the time constants, θ_*c*_ and θ_*v*_ are the time delays caused by finite speed of neural transition and secretion of noradrenaline, and $k_{c}^{S}$, $k_{v}^{S}$, and *k*_*v*_ are the transfer coefficients. Changes of concentration of noradrenaline and activity of parasympathetic control affect the heart rate through the sympathetic factor *f*_*s*_(*t*) and parasympathetic factor *f*_*p*_(*t*):

(14) $$f_{s}\,\left(t\right)=1+\left(C_{\varphi }^{s}+k_{\varphi }^{c}\right)\,\left(c_{c}\,\left(t\right)+\left({\hat{c}}_{c}-c_{c}\,\left(t\right)\right)\,\frac{c_{c}^{n_{s}}\,\left(t\right)}{{\hat{c}}_{c}^{n_{s}}+c_{c}^{n_{s}}\,\left(t\right)}\right),$$

$$f_{p}\,\left(t\right)=1+\left(C_{\varphi }^{p}+k_{\varphi }^{p}\right)$$

(15) $$\left(v_{p}\,\left(t-\theta_{p}\right)+\left({\hat{v}}_{p}-v_{p}\,\left(t-\theta_{p}\right)\right)\,\frac{v_{p}^{n_{p}}\,\left(t-\theta_{p}\right)}{{\hat{v}}_{p}^{n_{p}}+v_{p}^{n_{p}}\,\left(t-\theta_{p}\right)}\right)\,F\,\left(\varphi \,\left(t\right)\right),$$

where θ_*p*_ is the time delay, $C_{\varphi }^{s}$ and $C_{\varphi }^{p}$ are the parameters that reflect the influence of higher nervous centers on the parasympathetic and sympathetic factors of heart rate control, respectively, and $k_{\varphi }^{c}$, $k_{\varphi }^{p}$, ${\hat{c}}_{c}$, *n*_*s*_, ${\hat{v}}_{p}$, and *n*_*p*_ are the dimensionless factors. No separate equation was introduced to model the changes in concentration of acetylcholine (parasympathetic transmitter) because the rate of its secretion is faster than the dynamics on which the model is focused. The phase effectiveness curve represents the changes in the sinoatrial node sensitivity to parasympathetic control throughout the cardiac cycle:

(16) $$F\,\left(\varphi \right)=\varphi^{1.3}\,\left(\varphi -0.45\right)\,\frac{{\left(1-\varphi \right)}^{3}}{0.008+{\left(1-\varphi \right)}^{3}},$$

where φ is the phase of the cardiac cycle.

To simulate the sleep stages and awake state, we set the corresponding parameter values and generated long time series with the fixed parameters. To exclude the transient process, we did not analyze the first 1,000 s of time series. The length of the model time series was equal to the length of the experimental time series from the SIESTA database.

### Methods

The model data were compared with the experimental data from the SIESTA database using statistical measures, spectral analysis, and calculation of the largest Lyapunov exponent. The spectral analysis was carried out for the interbeat interval time series, which were extracted from the ECG signals by detecting the duration of time intervals between successive R-peaks. Since the dynamics of the heart in our model is described by a simple integrate-and-fire Eq. 1, in which the moment of sinoatrial node excitation corresponds to the reset of the cardiac cycle phase to zero, the location of R-peaks was defined by detecting the moment of such reset of the phase. We analyzed the spectral power in the ranges 0.05–0.15 Hz and 0.15–0.4 Hz to calculate the low-frequency (LF) and high-frequency (HF) indices, which reflect the activity of the sympathetic and parasympathetic control, respectively (Heart Rate and Variability., 1996).

Complexity is another important characteristic of CVS dynamics (Porta et al., 2017). To estimate the complexity, we calculated the largest Lyapunov exponent (Rosenstein et al., 1993) from the interbeat interval time series filtered in the range 0.05–0.4 Hz. The largest Lyapunov exponent was estimated using the Rosenstein algorithm (Rosenstein et al., 1993), which is well suited for the analysis of short time series. The first step was to find a nearest neighbor for each point of the reconstructed phase space, but the neighbors close in time were excluded from the analysis (Rosenstein et al., 1993). In dynamical systems, the average rate of divergence of the trajectories of the nearest neighbors obeys the following expression:

(17) $$\ln\left(L\right)\approx \ln\left(L_{0}\right)+\lambda_{0}\,t,$$

where *L*_0_ is the initial distance between the nearest neighbors, λ_0_ is the largest Lyapunov exponent, and *t* is the time of calculation. Then, λ_0_ is defined as follows:

(18) $$\lambda_{0}=\frac{\left\langle \ln\left(L\right)\right\rangle }{t}.$$

To reconstruct the phase space, we used the method of time delays. The dimension of the phase space was equal to 13. The time series were analyzed in windows having the length 1,000 s. A detailed explanation of the choice of this set of parameters is presented in Karavaev et al. (2019).

## Results

The mathematical model was used to simulate the healthy subjects in the awake state, during REM sleep, and stage 4 of non-REM sleep. The parameters of autonomic control were constant throughout all stages. We only changed the values of those parameters that were introduced to take into account the inputs from the higher nervous centers. The values of these parameters for each studied stage are presented in Table 1.

**FIGURE 2:**
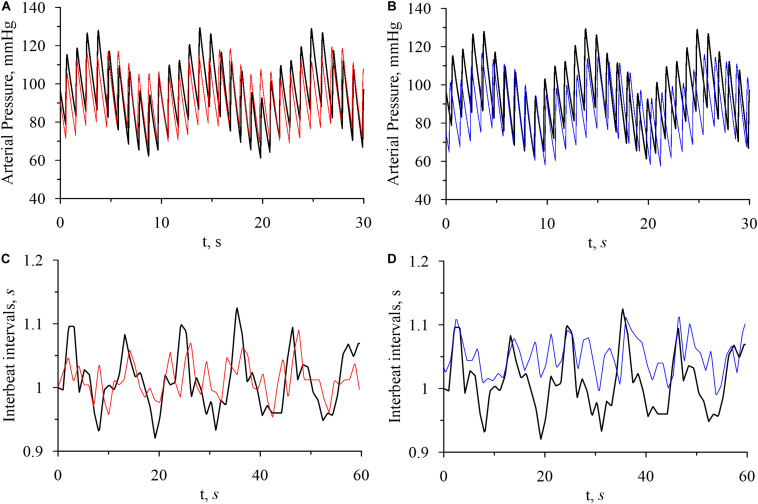
Model time series of arterial pressure and interbeat intervals. **(A)** Arterial pressure in the awake state (bold black line) and in REM sleep (thin red line). **(B)** Arterial pressure in the awake state (bold black line) and in non-REM sleep (thin blue line). **(C)** Interbeat intervals in the awake state (bold black line) and in REM sleep (thin red line). **(D)** Interbeat intervals in the awake state (bold black line) and in non-REM sleep (thin blue line).

Figure 2 shows the model and experimental time series of arterial pressure and interbeat intervals. It can be seen in Figure 2 that arterial pressure variability and interbeat interval variability are most pronounced in the awake state. These results agree well with the data from literature (Zemaityte et al., 1984; Heart Rate and Variability., 1996) and reflect the higher activation of sympathetic control during the wakefulness.

**FIGURE 3:**
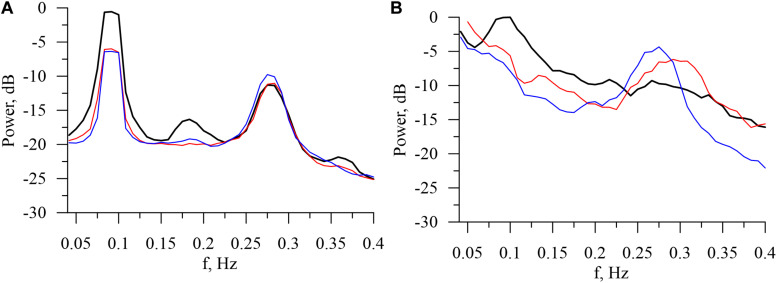
Logarithmic power spectra calculated from the model **(A)** and experimental **(B)** interbeat intervals in the awake state (bold black line), in REM sleep (thin red line), and in stage 4 of non-REM sleep (thin blue line).

The power spectra of model and experimental interbeat intervals are shown on Figure 3. In the model power spectra, the peaks at the frequencies of about 0.1and 0.29 Hz are significantly more narrow, sharp, and pronounced. This is explained by the fact that our model does not take into account the humoral control of CVS. However, the model qualitatively simulates the difference between the experimental spectra during wakefulness and at different stages of sleep. The power of the 0.1-Hz spectral component, which is associated with the sympathetic activation, takes the highest value in the awake state. The power of the 0.29-Hz component, which is associated with the parasympathetic control, takes the highest value during the stage 4 of non-REM sleep. These results are in good agreement with the known results of other studies (Zemaityte et al., 1984; Sommers et al., 1993).

The spectral and statistical indices calculated from model and experimental data are presented in Table 2.

**TABLE 2:** Spectral and statistical indices obtained from the model and experimental data (mean ± standard error of the mean).

**Index**	**Awake**		**REM sleep**		**Non-REM sleep**	
	**Experiment**	**Model**	**Experiment**	**Model**	**Experiment**	**Model**
HR, bpm	78 ± 6	60 ± 0.4	74 ± 4	59 ± 0.3	74 ± 2	57 ± 0.3
LF, ms^2^	841 ± 52	824 ± 10	451 ± 56	249 ± 7	187 ± 24	208 ± 5
HF, ms^2^	459 ± 47	234 ± 5	392 ± 46	224 ± 4	227 ± 33	279 ± 3
SP, mmHg	–	111 ± 1	–	110 ± 1	–	105 ± 1
DP, mmHg	–	75 ± 1	–	74 ± 1	–	68 ± 1
λ_0_	0.024 ± 0.001	0.019 ± 0.001	0.022 ± 0.005	0.022 ± 0.001	0.022 ± 0.001	0.022 ± 0.001

*HR, heart rate; LF, sympathetic activity index; HF, parasympathetic activity index; SP, systolic arterial pressure; DP, diastolic arterial pressure; λ_0_, the largest Lyapunov exponent.*

## Discussion

PNEUMA is one of the most famous models that simulate the influence of higher nervous centers on the dynamics of autonomic control during sleep stages and awake state (Cheng et al., 2010). The PNEUMA model takes into account many factors, such as respiration, chemoreceptors, and blood hydrodynamics. As a result, the PNEUMA model is high-dimensional and complex, which makes it difficult to interpret the results of model studies and observed effects. The high order of the model makes it almost impossible to reconstruct the model parameters from the data of a particular patient and to individually fit the model. The authors themselves stated that there is no available database that can be used to verify their model, and many parameters do not have physiological meaning (Cheng et al., 2010).

We have proposed a mathematical model that allows one to simulate the heart rate variability and arterial pressure oscillations under the influence of respiration and autonomic control loops. Earlier, it was shown that taking into account the non-linear properties and self-oscillatory dynamics of these loops it is possible to explain the experimentally observed complex chaotic dynamics of the heart rate and synchronization processes within the CVS (Ringwood and Malpas, 2000; Prokhorov et al., 2003; Karavaev et al., 2009, 2016, 2019; Kiselev et al., 2016a, 2020). In the present paper, a modified model is proposed that takes into account the influence of the activity of the cerebral cortex on autonomic control in the states of sleep and wakefulness. The respiratory process and some other factors are included into the model in a simplified form. Nevertheless, the proposed model allows us to simulate complex oscillatory dynamics of the interbeat intervals associated with the autonomic control of circulation.

For simplicity of the model, we have accepted a number of limitations: the model does not account for humoral control (Ganten and Stock, 1978; Chopra et al., 2011), blood hydrodynamics, and local control mechanisms (Clifford, 2011; Hong et al., 2020), such as the Bowditch effect (Usman et al., 2020). This explains the lower λ_0_ in the model compared to the experimental data. The narrower peaks in the model power spectrum compared to the experimental one (Figure 3) can be explained in a similar way. Moreover, the model is stationary, while real CVS is not.

Despite the aforementioned limitations, we consider the model to be an adequate representation of the dynamics of autonomic control during sleep and wakefulness. For experimental interbeat intervals, LF index, which is associated with the activity of the sympathetic autonomic control, typically takes the larger value in the awake state than in REM sleep and the smaller value in non-REM sleep than in REM sleep (Kantelhardt et al., 2002). The proposed model shows similar properties of the LF index. The model HF index, which is associated with the activation of the parasympathetic control, takes the largest value in the 4th stage of non-REM sleep. We obtained similar results for many experimental signals.

Our experimental data did not contain records of arterial pressure, but the known results (Sommers et al., 1993) report that both systolic and diastolic arterial pressure during REM sleep is slightly lower or the same as in the awake state, while during non-REM sleep, the arterial pressure is significantly lower. The results of our model agree well with this experimental observation.

Estimations of the largest Lyapunov exponent λ_0_ from the model and experimental data in both REM and non-REM sleep are in a good agreement. However, in the awake state, the estimations of λ_0_ differ for the model and experimental data. This disagreement is due to the simplicity of the model compared to the real system.

Some more compact models use stochastic components to simulate the elements of blood circulation (Ivanov et al., 1998; Kantelhardt et al., 2003). Such approaches may have advantages in the compactness of equations, but they do not allow one to study the non-linear dynamics of blood circulation and build personalized models.

In further research, we plan to develop a method for reconstructing the parameters of our model from experimental signals of ECG, blood pressure, and respiration. For example, the delay times of model equations can be estimated using different techniques (Prokhorov et al., 2005; Sysoev et al., 2016). However, to propose such a technique, it is necessary to have a good understanding of the capabilities and limitations of the model. In the present study, we took a step in this direction by taking into account the influence of brain activity on CVS during sleep and wakefulness. Such personalized mathematical model can be used in the future for the development of personalized medicine and will provide a new tool for the study of autonomic control.

## Conclusion

We have proposed a mathematical model of autonomic control in a form of fourth-order system of non-autonomous differential equations with time delay. Despite its compact structure, the model is able to simulate the dynamics of autonomic control during sleep stages and in awake state and related changes in interbeat intervals. The adequacy of the proposed model is demonstrated by comparing its time series with experimental records of healthy subjects in the SIESTA database.

## Data Availability Statement

The data analyzed in this study is subject to the following licenses/restrictions: These are data which belong to medical faculties and are not publicly available. Requests to access these datasets should be directed to TP (thomas.penzel@charite.de), he will provide a link to people at The SIESTA group in Vienna, Austria.

## Ethics Statement

For testing the model, it was compared to the experimental electrocardiogram (ECG) signals from the SIESTA database (Klösch et al., 2001). The studies involving human participants were reviewed and approved by the Ethics Committee of Klinikum der Philipps-Universität Marburg, Germany. The study participants, all above age of 18 years, provided written informed consent to participate in the study.

## Author Contributions

AK and TP finalized the manuscript for submission. All authors contributed to writing and discussion on the manuscript.

## Conflict of Interest

The authors declare that the research was conducted in the absence of any commercial or financial relationships that could be construed as a potential conflict of interest.
